# Modern contraceptive use and factors associated with use among postpartum women in Ethiopia; further analysis of the 2016 Ethiopia demographic and health survey data

**DOI:** 10.1186/s12889-020-08802-6

**Published:** 2020-05-12

**Authors:** Gizachew Worku Dagnew, Melash Belachew Asresie, Gedefaw Abeje Fekadu, Yared Mulu Gelaw

**Affiliations:** 1 Department of Reproductive Health and population studies, School of Public Health, College of Medicine and Health Science, Bahir Dar University, Bahir Dar, Ethiopia; 2 Department of Health Service Management, School of Public Health, College of Medicine and Health Sciences, Bahir Dar University, Bahir Dar, Ethiopia

**Keywords:** Postpartum, Contraceptive, Ethiopia, EDHS

## Abstract

**Background:**

The postpartum period is a critical time to improve maternal and child health. It is a time for accessing contraceptives to prevent short inter-pregnancy intervals. More than 95% of postpartum women do not want to get pregnant within 12 months. However, many women in Ethiopia experience an unintended pregnancy, and there is low information about postpartum contraceptive use among women who have family planning demand. Therefore, this study aimed to estimate the prevalence of postpartum contraceptive use and its predictors among women who give birth 12 months before the survey in Ethiopia.

**Methods:**

We used the 2016 Ethiopia demographic health survey data for this analysis. The survey was a community-based cross-sectional study conducted from January 18 to June 27, 2016. The survey employed a two-stage stratified cluster sampling technique. A total of 2304 postpartum women were included. Bivariate and multivariable logistics regressions were done to identify factors associated with postpartum contraceptive use. A *p*-value < 0.05 was used to declare statistical significance.

**Results:**

About 23.7% (23.7, 95% CI: 20.7–27.0%) of postpartum women were using modern contraceptives. Women who were urban residents (AOR = 2.18; 95%CI: 1.34–3.55), those who attended secondary or higher education (AOR = 1.79; 95%CI: 1.04–3.10), women who attended 1–3 (AOR = 2.33; 95%CI:1.27–4.25) or 4 or more ANC visits (AOR = 2.59; 95%CI:1.43–4.69) and women who delivered at a health facility (AOR = 1.86; 95%CI: 1.23–2.81) had higher odds of modern contraceptive use during the postpartum period. Similarly, women who reported the last child was no more wanted (AOR = 1.83; 95%CI: 1.01–3.31), women who decided for contraceptive use (AOR = 2.03; 95%CI: 1.13–3.65) and women whose recent child was male (AOR = 1.38; 95%CI: 1.01–1.88) had higher odds of modern contraceptive use.

**Conclusion:**

Postpartum contraceptive use was low in Ethiopia. Strengthening health facility delivery, promoting girls’ education and encouraging women’s participation in deciding for contraceptive use would improve the uptake of modern contraceptives use during the postpartum period.

## Introduction

Postpartum family planning (PPFP) prevents unintended and closely spaced pregnancies [1]. According to the updated world health organization (WHO) medical eligibility criteria, postpartum and breastfeeding mothers can use progesterone-only pills, implants, the levonorgestrel-releasing IUD, condom/spermicides, male and female sterilization, emergency contraceptive, and diaphragm/cervical cap. The breastfeeding mothers can also use lactational amenorrhea until 6 months of delivery and combined oral contraceptives after 6 months of delivery [2–4].

Less than 5% of postpartum women in low-income countries wished to have another pregnancy within 12 months. But a large number of women had a high level of unmet need for contraceptives [5]. Unmet need for family planning (FP) is defined as women who are not pregnant and not postpartum amenorrhoeic and are considered fecund and want to postpone their next birth for two or more years or stop childbearing altogether but are not using a contraceptive method [6].

A survey in 21 low and middle-income countries indicated that 61% of postpartum women had an unmet need for family planning. The survey added that more 50% of second births occurred within a short inter-pregnancy interval [7]. About 20% of births in Sub-Saharan Africa occurred with an interval of below 24 months [8].

Short inter-pregnancy interval has a great impact on the child and maternal health. Children born with short-birth intervals (less than 24 months) are at a higher risk of mortality and undernutrition. Similarly, mothers with short birth intervals are at a higher risk of miscarriage, pre-eclampsia, uterine rupture and other maternal complications [8–13]. The postpartum period is a critical time to address the unmet need for family planning and reduce the risks of closely spaced pregnancies. Spacing pregnancies at least 2 years apart can avert about 10% of infant deaths and 21% of child deaths [14].

Although 95% of women intend to postpone pregnancies for at least 2 years, modern contraceptive use at the postpartum period varies across countries and range from 73.5% in Zambia to 4% in Pakistan [5, 15, 16]. Studies in Ethiopia showed that modern contraceptives use in the postpartum period range from 80.2% in Addis Ababa [17] to10.2% in Dabat [18]. Mothers who attended antenatal care (ANC), those who delivered in the health facility, those women with a higher level of education, urban residents, and women with a history of contraceptive use before the index pregnancy were predictors of postpartum contraceptive (PPC) use.

Currently, Ethiopia is implementing different programs to increase postpartum contraceptive use. The national family planning guideline recommends integrating FP counseling during ANC, delivery and post-natal care (PNC) services [19]. Postpartum women who choose a method during antenatal care or at the time of delivery receive high-quality postpartum family planning services before discharge [20]. Yet, the unmet need for contraceptives is high and many pregnancies have short inter-pregnancy intervals [7].

Although there is an improvement in modern contraceptives use among married reproductive-age (15–49 years) women, unmet need is still high. Consequently, there are high numbers of undesired births, short inter-pregnancy interval and neonatal mortality [6]. Furthermore, there is little information about PPC use, especially among women who have a demand for family planning. Also, most published research works did not include potential predictor variables like knowledge on ovulation, women participation for decision making, birth interval and wantedness of the last-child.

Therefore, this analysis was done to assess the prevalence of postpartum contraceptive use and its predictors among women who have a demand for family planning in Ethiopia. The findings of this study can help to design an effective strategy to prevent unintended pregnancy and short inter-pregnancy interval.

## Methods

### Data

We used the 2016 EDHS data, collected from January 18 to July 27, 2016, from all administrative regions. It was a community-based cross-sectional survey. A total of 15,683 reproductive age (15–49 years) women were included in the survey. The 2016 EDHS used a two-stage stratified cluster random sampling technique to ensure the representativeness of the sample by regions and residence. The survey covered all administrative regions. Initially, each region was stratified into urban and rural areas yielding 21 sampling strata. After stratification, a total of 645 enumeration areas (202 in urban areas and 443 in rural areas) were selected with probability proportional to enumeration are size based on the 2007 Ethiopia population and housing census. A household listing operation was done from September to December 2015. Then, 28 households from each cluster were selected using a systematic random sampling technique [6].

**Fig. 1:**
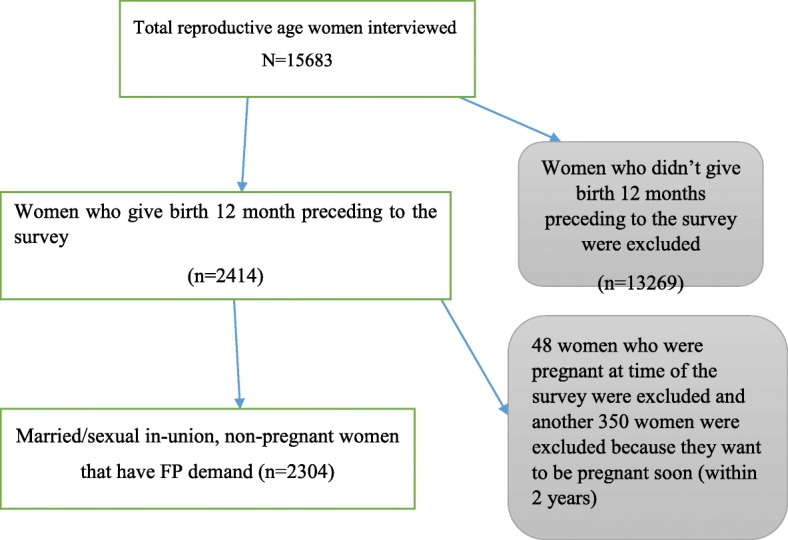
Schematic presentation to select women included to study postpartum family planning use and factors associated with use from the 2016 Ethiopia Demographic and Health Surveys

This analysis included all women who were married/in sexual union, non-pregnant, and gave birth within 12 months of the survey. Women who have a desire for childbirth within 2 years were excluded from the analysis. Based on these criteria, a total of 2304 women were included in the final model (Fig. 1).

### Measurements

#### Outcome variable

The outcome of this analysis was postpartum contraceptive use; a variable with two outcomes (yes/no). It was measured based on the woman’s self-report of modern contraceptive use at the time of the survey (current use). Modern contraceptive methods reported in this study include; female sterilization, implant, intrauterine device (IUD), injectable, oral contraceptive, emergency contraceptive, condom, lactational amenorrhea and periodic abstinence [6].

### Independent variables

#### The socio-demographic variables

nclude women age (15–24, 25–34 and ≥ 35 years), residence(urban and rural), religion (Christian, Muslim and others), women’s education status (no formal education, primary, and secondary or above), employment status (working and not working), region (Tigray, Afar, Amhara, Oromia, Somali, Benshaguel, South Nation Nationality People republic (SNNPR), Gambella, Harari, Addis Ababa, and Dire Dawa) and wealth index (poor, middle and rich).

#### Reproductive health and related variables

Include; the number of under-five children in the households (one and more than one), number of living children (one, 2–4 and ≥ 5), birth order (first, 2–4, and ≥ 5), preceding birth interval (≤ 24 months, ≥ 25 months and no previous birth), wantedness of the last child (wanted, wanted later, and wanted no more), sex of index child (male and female), duration after childbirth (1–2 months, 3–6 months, and 7–12 months), participation on contraceptive use decision (yes/no), visited by a health worker (yes/no), and exposure to family planning messages on mass media (newspaper, radio, television, or mobile SMS text) (yes/no).

#### The maternal health service use related variables

include ANC attendance (no ANC, 1–3 ANC and ≥ 4 ANC), place of delivery (health facility and home) and PNC attendance (yes/no).

#### PNC attendance

Postnatal check includes women who received at least one PNC check from a doctor, midwife, nurse, or health extension worker after delivery or after discharge/delivery at home before 42 days of delivery [6]. This was measured based on the woman’s response to a question of whether she had this visit or not.

#### Women participation in decision making for contraceptive use

women were considered as participated when the women reported that she decided contraceptive use by herself or jointly with her husband [6].

### Statistical analysis

We used STATA 14.0 for this analysis. Descriptive statistics were calculated for all variables. Data were weighted to ensure the representativeness of the survey results at the regional level and by residence, and to account for non-response. Furthermore, the analysis was adjusted to account for the complex survey design and robust standard errors (stratification and clustering) using the ‘svy’ command in Stata.

Bivariate logistics regression analysis was conducted to select the candidate variable for multivariable logistics regression. Variables with a *p*-value ≤0.2 in the binary logistic regression analysis were included in the multivariable logistic regression model. Multicollinearity between each explanatory variable was checked by using the ‘pwcorr’ command in Stata. Model fitness was checked by using the Hosmer Lemeshow’s model good fit (*p* > 0.05) [21].

The multivariable analysis was done to identify factors associated with postpartum contraceptive use. Only variables with a *p*-value of less than 0.05 in the multivariable analysis were considered statistically significant. The adjusted odds ratios (AOR) with its corresponding 95% confidence interval were presented in the results section.

## Results

### Socio-demographic characteristics of women

A total of 2304 reproductive-age (15–49 years) women who gave birth within 12 months of the survey were included in the analysis. The mean age (±sd) of the study participants was 28.0 (±6.6) years. Almost half (49.0%) of them were aged 25–34 years. About 88% of women were rural residents. Nearly 60% of participants did not attend formal education. More than 90% of participants were from major regions (Oromia 44.7%, Amhara 19.7%, SNNPR 20.5% and Tigray accounts 7.4%), 4.6% of participants were from developing regions (2.4% Somali, 1.1% Benshangul, 0.7 Afar, Harari ad Gambela accounts each 0.2%), and the remaining 3% comes from two city administration (2.6% Addis Ababa, 0.4% Diredawa) (Table 1).

**Table 1:** Sociodemographic, Reproductive health and maternal health service use characteristics of married/ in union postpartum women in Ethiopia, 2016 EDHS

Variables (*n* = 2304)	Categories	Number (%)
Age in years	15–24	735 (31.9)
	25–34	1129 (49.0)
	≥35	440 (19.1)
Residence	Urban	286 (12.4)
	Rural	2018 (87.6)
Wealth index	Poor	1010 (43.9)
	Middle	482 (20.9)
	Rich	811 (35.2)
Educational status	No formal education	1330 (57.7)
	Primary	769 (33.4)
	Secondary or above	205 (8.9)
Religion	Christian	1287 (55.8)
	Muslim	960 (41.6)
	Others	58 (2.5)
Working status	Not working	1410 (61.2)
	Working	894 (38.8)
Number of living children	One	523 (22.7)
	2–4	1044 (45.3)
	5+	737 (32.0)
Preceding birth interval	≤24 months	286 (12.4)
	≥25 months	1519 (65.9)
	No preceding birth	498 (21.6)
Birth order of current child	First	495 (21.5)
	2–4	976 (42.3)
	5+	834 (36.2)
Women had participated in deciding for FP use	No	356 (15.5)
	Yes	1948 (84.5)
ANC attendant	No ANC	710 (30.8)
	1–3 ANC	775 (33.6)
	4+ ANC	820 (35.6)
Place of delivery	Home	1366 (59.3)
	Health facility	938 (40.7)
Attending at least one Postnatal visit	No	1826 (79.2)
	Yes	479 (20.8)
Duration of postpartum	1–2 months	593 (25.7)
	3–6 months	1033 (44.8)
	7–12 months	678 (29.4)
Media Exposure for FP messages	No	1714 (74.4)
	Yes	590 (25.6)

### Reproductive health characteristics and maternal health service use

Seven-hundred thirty-seven (32.0%) study participants had five or more living children. One-hundred eighty-eight (40.6%) participants want no more children in the future. Two-hundred eighty-six (12.4%) births had a short inter-pregnancy interval (≤ 24 months). Eight-hundred thirty-four (36.2%) of the index child was fourth or more in terms of birth order. About 1138 (49.4%) of the index child was male sex. Sixty-nine percent of women reported that they attended at least one ANC visits and 40.7% of women reported that they delivered at health facilities. One-third (34%) of the study participants were visited by field workers in the last 12 months, ad 66.8% of women report that they visited the health facility in the same period (Table 1).

### Postpartum contraceptive use

**Fig. 2:**
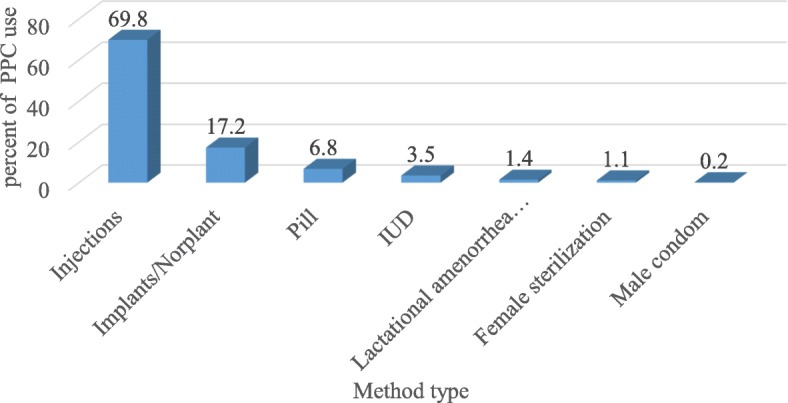
Contraceptive method mix among postpartum women in Ethiopia; EDHS 2016

The analysis identified that 23.7% (95%CI: 20.7–27.0%) postpartum women were using modern contraceptive methods at the time of the survey. The most common contraceptive used were injectable (69.8%), followed by implants (17.2%), and the least frequent contraceptive method used was male condom (0.2) (Fig. 2).

### Postpartum contraceptive use by background characteristics

**Fig. 3:**
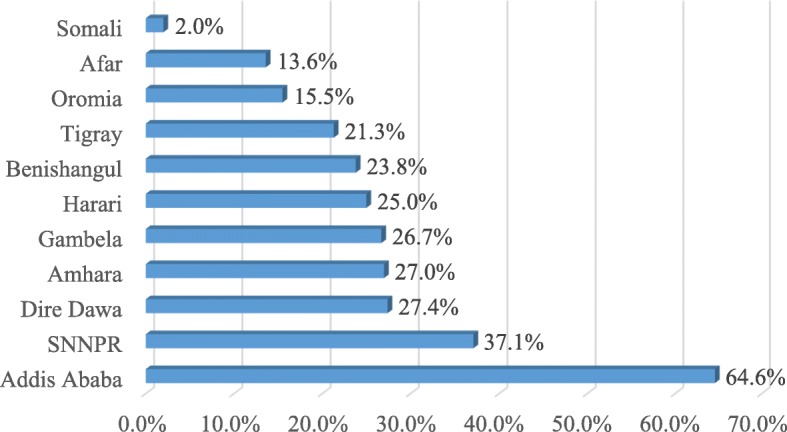
Postpartum contraceptive use by region in Ethiopia; EDHS 2016

Postpartum contraceptive use varied by region. The level of use was lowest in Somali (2%) and Afar (13.6%) regions. The highest level of use was in Addis Ababa (64.6%) and SNNPR (37.1% (Fig. 3).

The level of postpartum contraceptive use was relatively lower among women aged more than 35 years compared to those aged 15–24 years. Similarly, postpartum contraceptive use was higher among women with few numbers of children compared to those with a high number of children. For example, postpartum contraceptive use among women with only one living child was 32.2% compared to 13.7% among women who had more than five children. On the other hand, postpartum contraceptive use was relatively higher among rich women compared to the poor (Table 2).

**Table 2:** Factors associated with PPC use among married/in-union women in Ethiopia; EDHS 2016

Variables (*n* = 2304)	Current FP use		COR	95%CI	AOR	95%CI
	Yes (%)	No(%)				
**Age**						
15–24	28.2	71.8	1.90*	1.17–3.08	1.41	0.80–2.49
25–34	23.4	76.6	1.48	0.91–2.40	1.11	0.65–1.87
35+	17.1	82.9	1		1	
**Place of residence**						
Rural	19.7	80.3	1		1	
Urban	52.1	47.9	4.43***	3.03–6.49	2.20**	1.35–3.60
**Educational status**						
No formal education	16.2	83.8	1		1	
Primary	30	70	2.21***	1.58–3.11	1.41	0.96–2.05
Secondary ad above	49.3	50.7	5.03***	3.20–7.93	1.81*	1.04–3.12
**Wealth index**						
Poor	15.1	84.9	1		1	
Middle	22.4	77.6	1.63*	1.06–2.49	1.38	0.87–2.19
Reach	35.3	64.7	3.07***	2.11–4.47	1.46	0.94–2.27
**No. of ANC visit**						
No ANC visit	8.9	91.1	1		1	1
1–3 ANC	26.1	73.9	3.60***	2.10–6.18	2.33**	1.28–4.24
4 + ANC	34.3	65.7	5.32***	3.13–9.03	2.61**	1.44–4.72
**Place of delivery**						
Health facility	37.3	62.7	3.54***	2.57–4.86	1.85**	1.22–2.81
Home	14.4	85.6	1		1	
**Received PNC services**						
No	20.4	79.6	1		1	
Yes	36.2	63.8	2.21***	1.61–3.03	0.87	0.58–1.31
**Sex of the last child**						
Male	26.4	73.6	1.34*	1.01–1.77	1.38*	1.01–1.88
Female	21.1	78.9	1		1	
**Wanted status of last-child**						
Wanted soon	22.2	77.8	1		1	
Wanted later	29.6	70.4	1.48	0.99–2.20	1.81**	1.19–2.77
Wanted no more	23.4	76.6	1.07	0.63–1.82	1.83*	1.01–3.31
**Duration of postpartum**						
1–2 months	23.3	76.7	1		1	
3–6 months	22.2	77.8	1		1.09	0.72–1.64
7–12 months	26.3	73.7	1.23	0.90–1.67	1.10	0.71–1.71
**Media exposure for FP messages**						
No	20.8	79.2	1		1	
Yes	32.3	67.7	1.82**	1.30–2.55	0.88	0.60–1.30
**Participation in FP use**						
No	14.2	85.8	1		1	
Yes	25.4	74.6	2.06**	1.22–3.48	2.01*	1.14–3.55
**Visited by fieldworker in the last 12 months**						
No	20.6	79.4	1		1	
Yes	30	70	1.66**	1.19–2.31	1.39	0.98–1.97

*** *p* < 0.001, ** *p* < 0.01, * *p* < 0.05, *COR* Crude Odd Ratio, *CI* Confidence Interval, *AOR* Adjusted Odd Ratio

Postpartum contraceptive use was also higher among women who delivered at health facilities (37.3%) compared to women who delivered at home (14.4%). Similarly, PPC use was relatively high among women who attended ANC (34.3%) compared to those who did not attend ANC (8.9%).

### Factors associated with postpartum contraceptive use

On multivariable logistics regression, residence, educational status, place of delivery, ANC attendance, participation on FP use, wantedness and sex of the last child were statistically significant at *p*-value < 0.05. Women who attended secondary or higher education had higher odds (AOR = 1.81; 95%CI: 1.04–3.12) of postpartum contraceptive use compared to women who did not attend formal education. Urban women had higher odds (AOR = 2.20; 95%CI: 1.35–3.60) of PPC use compared to rural women. Women who delivered at health facility had higher odd (AOR = 1.85; 95%CI: 1.22–2.81) of PPC use compared to those who delivered at home. Similarly, women who attend 1–3 ANC visits (AOR = 2.33; 95%CI: 1.28–4.24) or 4 or more ANC (AOR = 2.59; 95%CI: 1.44–4.72) visits had high odds of PPC use compared to those who didn’t have ANC visit. Similarly, the odd of postpartum contraceptive use were higher among women whose recent birth was wanted later (AOR = 1.81; 95%CI: 1.19–2.77) and no more wanted (AOR = 1.83; 95%CI: 1.01–3.31) compared to women whose recent birth was wanted soon (Table 2).

## Discussion

The analysis included women who did not intend to have children soon. That means, only women who had a demand for modern contraceptives were included in this analysis. But only 23.7% of Ethiopian women were using modern contraceptives during the postpartum time. The implication is that most Ethiopian women were exposed to unintended pregnancy during the postpartum period. The level of use was lower than the study in Kenya (86.3%) [22] and other small scale studies in Ethiopia; Hossana (72.9%), Dessie (54.7%) and Addis Ababa [17, 23, 24]. The reason for the low level of postpartum contraceptive use in this study compared to other studies might be the difference in the educational status of the study participants. Almost all women in the Kenyan study attended formal education [22]. In the Hossana, Dessie and Addis Ababa studies, 68–89% of the participants attended formal education [17, 23, 24] compared to only 42.3% in this study. Education can increase women’s awareness and level of understanding about the risk of being pregnant in the postpartum period. The other reason for the lower use in this study might be the difference in health facility delivery. In the current study, only 40.7% of women delivered at a health facility, but more than 80% of women who participated in the above studies delivered in health facilities [17, 23, 24]. Women who delivered at the health facilities are more likely to be counseled about postpartum family planning and initiated to use a contraceptive.

More than three-fourths of women reported using short-acting contraceptive methods (69.8% injectable, and 6.8% pills were the predominant methods). This may be related to women’s preference for short-acting methods, provider bias or limited access to long-acting methods [25]. These dominant types of contraceptive use were observed in different studies, especially in low and middle-income countries [26–29].

The current study revealed that education is positively associated with postpartum contraceptive use. The odds of contraceptive use was higher among women who attended secondary or higher education compared to those who did not attend. This finding was in line with studies done in Uganda and other studies in Ethiopia [5, 16, 23, 24]. The reason for this might be that educated women can understand the risk of a short inter-pregnancy interval and the benefits of contraceptive use during the postpartum period.

The study showed that urban women had higher odds of modern contraceptive use in the postpartum period compared to their rural counterparts. The reason for this might be that urban women have better access for maternal health services including contraceptives compared to rural women. This finding was in line with a study in Northwest Ethiopia [18].

This analysis showed that ANC attendance was positively associated with postpartum contraceptive use. The possible explanation for this is that women who attend ANC were more likely to be counseled about postpartum contraceptive use. Such counseling and information may have increased women’s knowledge for family planning and subsequent uptake of contraception during the postpartum period [30]. This finding was in line with studies from Mexico, Nigeria, Ghana and Gondar [29, 31–33]. Similarly, women who delivered at a health facility had higher odds of postpartum contraceptive use compared to women who delivered at home. The possible explanation for this finding might contraceptive information and initiation for women who delivered at a health facility. FP services are integrated with the delivery service in Ethiopia. This is indicated in the Ethiopian family planning guideline [19]. This information provision and counseling before discharge may increase the subsequent uptake of contraceptives [34]. This finding is consistent with the studies done in different countries including Ethiopia [5, 16, 18, 35, 36].

Wantedness of the recent child was another factor associated with postpartum contraceptive use. Mothers who reported their recent birth was wanted later and not wanted at all had high odds of postpartum contraceptive use compared to women who reported their recent child was wanted at the time of the survey. The implication is that women who achieved or surpassed the desired number of children were more likely to use contraceptives during the postpartum period. This implies that women who experienced unwanted birth are more likely to use contraceptives during the postpartum period to prevent additional unwanted children [22]. This finding was consistent with the study done in Nepal [37].

Women who decided contraceptive use by themselves or jointly with their partner were more likely to use contraceptives during the postpartum period. This is related to women’s autonomy. This finding was supported by findings from other studies [38–40].

This study showed that the sex of the recent child was significantly associated with postpartum contraceptive use. Women whose recent child was male had higher odds of postpartum contraceptive use. This might be due to sex preference [41, 42]. This is supported by studies done in China, Nepal, India, and other global context studies [43–46].

## Conclusions

Postpartum contraceptive use was low in Ethiopia. More than three-fourths of women have had a risk of facing mistimed or unwanted pregnancy. Urban women, women who attended secondary or higher levels of education, women who attended ANC or delivered at health facilities and women with unintended birth had higher odds of postpartum contraceptive use. Similarly, women who participated in a decision regarding contraceptive use and women whose recent child was male had higher odds of postpartum contraceptive use. Strengthening skilled maternal health services use is recommended to increase postpartum contraceptive uptake. Additionally, promoting girls’ education, and empowering women to participate in deciding for contraceptive use are recommended to increase postpartum contraceptive use.

## Data Availability

The datasets used for this study are available from the DHS program website [http://dhsprogram.com/data/] upon permission.

## Abbreviations

ANC: Antenatal Care
AOR: Adjusted Odds Ratio
CI: Confidence Interval
EDHS: Ethiopian Demographic and Health Survey
FP: Family Planning
IUD: Intrauterine Device
PPC: Postpartum Contraceptive
